# Evaluation of Jaw Osteonecrosis Following Intra-Ligament Anesthesia in Zoledronate-Treated Rats: a Histological Evaluation

**DOI:** 10.30476/DENTJODS.2022.91452.1583

**Published:** 2023-03

**Authors:** Saeed Moradi, Bibi Marjan Razavi, Siavash Moushekhian, Narges Pourmahmoud, Mohammad Marvi

**Affiliations:** 1 Dental Material Research Center, Dept. of Endodontics, Faculty of Dentistry, Mashhad University of Medical Sciences, Mashhad, Iran; 2 Targeted Drug Delivery Research Center, Pharmaceutical Technology Institute, Dept. of Pharmacodynamy and Toxicology, School of Pharmacy, Mashhad University of Medical Sciences, Mashhad, Iran; 3 Dental Research Center, School of Dentistry, Mashhad University of Medical Sciences, Mashhad, Iran; 4 Pharmacist, Mashhad, Iran; 5 Dept. of Endodontics, School of Dentistry, Mashhad University of Medical Science, Mashhad, Iran

**Keywords:** Bisphosphonates, Zoledronic Acid, Bisphosphonate-Associated Osteonecrosis of the Jaw

## Abstract

**Statement of the Problem:**

One of the rare adverse effects in patients who take bisphosphonates is the osteonecrosis of the jaw in the oral cavity following any trauma such as tooth extraction.

**Purpose:**

The aim of this study is the histopathological evaluation of the jaw following intra-ligament anesthesia injection in Zoledronate-treated rats.

**Materials and Method:**

In this descriptive-experimental study, rats weighing 200-250 g were divided into 2 groups. The first group received a 0.6 mg/kg dose of zoledronate and the second group received normal saline. Five injections with a 28-day interval were performed. At the end of the injection, the animals were sacrificed. Then, five-micrometer histological slides were prepared from the first maxillary molars and the surrounding tissues. Hematoxylin and eosin staining was performed to evaluate osteonecrosis, infiltration of inflammatory cells, fibrosis, and root and bone resorption.

**Results:**

There was no difference between the macroscopic and clinical features in both groups and no evidence of osteonecrosis of the jaw was observed in the samples. From the histological point of view, all the samples had normal tissues and none of them showed any evidence of inflammation, tissue fibrosis, disorder, or pathological root resorption.

**Conclusion:**

According to the histological findings, the periodontal ligament space, the bone adjacent to the roots, and the dental pulp conditions were similar in both groups. Osteonecrosis of the jaw did not develop in the rats that took bisphosphonates after intraligamental injection.

## Introduction

As a group of antiresorptive agents, bisphosphonates are used in bone diseases with high bone resorption such as bone malignancy (to prevent metastasis), osteoporosis, multiple myeloma, Paget’s disease, and hypercalcemia [ 1 ]. These drugs induce apoptosis in osteoclasts and inhibit their activity. Moreover, they decrease bone resorption and increase bone mineralization [ 2 ]. Bisphosphonates have a high affinity for the skeleton and accumulate in active bone remodeling sites [ 3 ]. They remain in the bone for a long time and have detrimental effects on it. Zoledronate is a highly potent drug and belongs to the third generation of bisphosphonates [ 4 ]. The osteonecrosis of the jaw is one of the rare adverse effects of these drugs and can happen after traumatic procedures such as dental extraction and maxillofacial surgery [ 5 ]. One of the important advancements in dental therapeutics is the development of safe and effective local anesthetic techniques. However, anesthetic effectiveness is sometimes poor especially after a mandibular or maxillary nerve block in patients with severe inflammation in the dental pulp [ 6 ]. For these patients, some supplemental anesthetic techniques are available. In intra-ligament anesthesia, a high injection pressure is used to force the local anesthetic solution through the periodontal ligament into the cancellous medullary bone surrounding the tooth [ 7 ]. 

Intra-ligament anesthesia may cause injury and trauma to the periodontal ligament and the surrounding tissues. Hence, the aim of this study was a histological evaluation of intra-ligament injection in zoledronate-treated rats to assess the osteonecrosis of the jaw in them.

## Materials and Method

The protocol of this study was reviewed and approved by the Ethics Committee of Mashhad University of Medical Sciences (Number: 911155). The institutional and national standards for the care and use of laboratory animals were followed. The current experiment complied with the ARRIVE guidelines. A total of 20 male Wistar rats (mean age: 140 days; weight: 200-250 g) were obtained from the animal house of the university and acclimatized for 10 days prior to the experiment. The animals were housed in a temperature- and humidity-controlled environment with food and water supplies. The rats were randomly allocated to two groups (10 rats per group). Group 1 received intraperitoneal (IP) infusion of zoledronate (Zometa, Novartis Pharma, Basel, Switzerland) at the dose of 0.6 mg/kg every 28 days for 5 months [ 8 ].

Group 2 (control) received the same volume of normal saline every 28 days for 5 months. All injections were performed by one of the researchers (N P). Forty-five days after saline/zoledronate infusion, all the rats in both groups underwent the infiltration injection of lidocaine with 1/100000 epinephrine in the first molar in the right side of the upper jaw with a 20-gauge needle and intra-ligament injection with a special syringe (with a 20-gauge needle) in the first molar in the left side in the mesiopalatal angle. Intra-ligament injection was performed with back pressure in the periodontal ligament space so that the color of the surrounding tissues turned white after injection. All anesthesia injections were performed by one of the researchers (S M).

One hundred fifty days after the start of the study, the animals were subjected to vital perfusion and the first molar teeth and the surrounding tissues were separated from the maxilla. First, the samples were fixed in formalin 10% for 24 hours. Afterwards, calcification was performed with 85% formic acid for one week and the samples were embedded in paraffin. Subsequently, 5-µm-thick sections were prepared and used for hematoxylin and eosin staining. A histological examination was performed to evaluate osteonecrosis, infiltration of inflammatory cells, fibrosis, and root and bone resorption.

## Results

**Figure 1:**
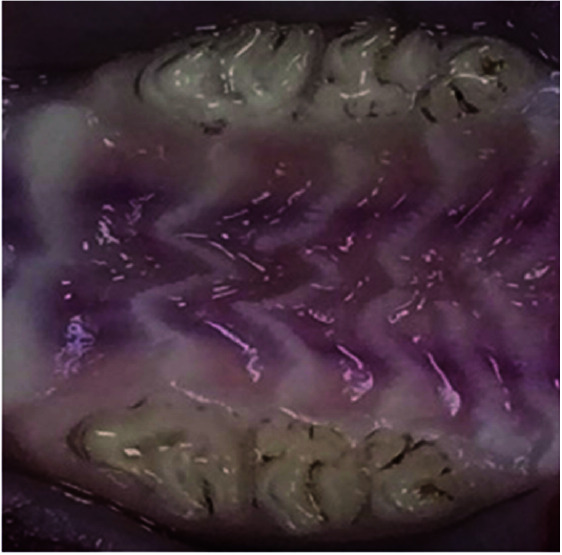
The macroscopic image of the maxilla of the rats treated with zoledronate and intra-ligament injection

**Figure 2:**
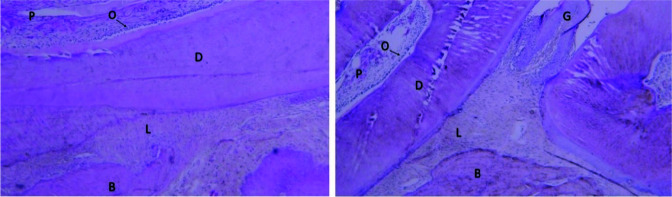
The histological view of the zoledronate-treated group. Right: Intra-ligament injection subgroups (40×); Left: Infiltration injection subgroups (40×) (B: Bone; D: Dentin; G: Gingiva; L: Periodontal ligament; O: Odontoblastic layer; P: Pulp)

**Figure 3:**
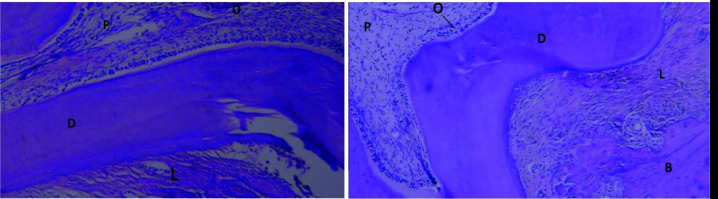
The histological view of the periodontal ligament and bone surrounding the tooth. Right: The zoledronate-treated group (40×); Left: The control group (40×) (B: Bone; D: Dentin; L: Periodontal ligament; O: Odontoblastic layer; P: Pulp)

**Figure 4:**
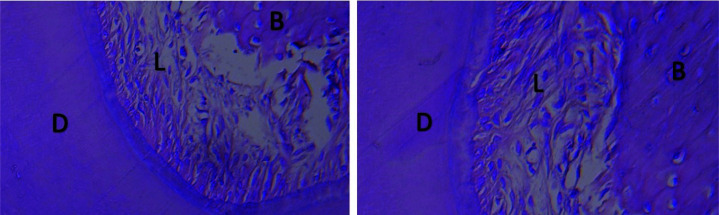
The histological view of the periodontal ligament space. Right: The zoledronate-treated group (100×); Left: The control group (100×) (B: Bone; D: Dentin; L: Periodontal ligament space)

**Figure 5:**
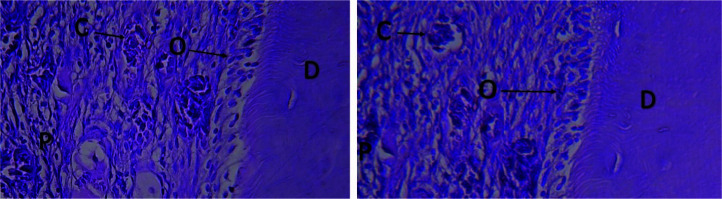
The histological view of the normal pulp and odontoblast cells. Right: The zoledronate-treated group (100×); Left: The control group (100×) (C: Capillary; D: Dentin; O: Odontoblastic layer; P: Pulp)

The histological evaluation was performed for the two groups (zoledronate and saline therapies). There was no evidence of wound and defect in the maxilla of the rats that received zoledronate and intra-ligament injection (Figure 1). There were no inflammatory cells in the histological views of the dental pulp, dentin, periodontal ligament, and bone in the two groups as well as in the intra-ligament and infiltration injection subgroups (Figure 2). The periodontal ligament and bone surrounding the roots had normal conditions, no differences were observed between the two groups, and subgroups (Figures 3 and 4). The periodontal ligament space was identical and normal in the two groups (Figure 3). The odontoblasts and dental pulp were normal in the two groups (Figure 5).

## Discussion

Bisphosphonates are non-metabolic drugs and pyrophosphate analogues, which prevent the transition of amorphous calcium to hydroxyapatite and the accumulation of calcium phosphate crystals [ 9 ]. Their most important clinical effect is the prevention of bone resorption. In their non-metabolized form, bisphosphonates remain in the bone over a long period of time. The activity of osteoclasts is reduced following the administration of these drugs [ 9 ]. One of the rare adverse effects of bisphosphonates is the osteonecrosis of the jaw, which can occur following injuries in the jaw such as tooth extraction [ 10 ]. Intraosseous injection may cause osteonecrosis of the jaw in patients who take bisphosphonates [ 10 , 13 ]. Incomplete anesthesia is one of the complications in the process of anesthesia and requires the use of supplemental anesthetic techniques. The intra-ligament anesthetic technique is a common supplemental anesthetic method [ 10 - 13 ]. Therefore, this study has been organized to investigate the relationship between bisphosphonate-related osteonecrosis of the jaw and intra-ligament injection. Although intra-ligament injection in the periodontal ligament space results in a suitable numbness, it has some adverse effects including decreased local blood flow, periodontal tissue destruction, and localized inflammation [ 11 - 13 ].

In the present study, no differences were observed in the histological samples of the treated and control groups. In addition, no sign of fibrosis was observed in the histological samples. On the other hand, *in vitro* studies have shown that zoledronate decreases the cell viability of both periodontal ligament fibroblasts [ 14 - 16 ] and tumor cells [ 17 - 18 ]. Allam *et al*. [ 19 ] reported that zoledronate had toxic effects on dental pulp cells in micro-molar concentrations, reducing their proliferation. The onset of the bisphosphonate-related osteonecrosis of the jaw may be due to dental pulp necrosis. 

This study revealed no differences between the two groups. The significant difference between the *in vitro* and *in vivo* results is due to the different conditions. Bisphosphonates reduce bone remodeling; prevent osteoclast activity, decrease bone loss, and increase bone density [ 20 - 21 ].

Pourgonabadi *et al*. [ 22 ] showed that zoledronate has anti-proliferative and pro-apoptotic effects in the dental pulp stem cells (DPSCs), which may lead to zoledronate-induced osteonecrosis. Furthermore, Pourgonabadi *et al*. [ 23 ] demonstrated that the long-term effects of alendronate decreased cell proliferation and apoptosis in DPSCs, resulting in the initiation or potentiation of alendronate-induced osteonecrosis. In pharmacokinetic studies, the serum levels of zoledronate are up to 1 μmol/L which is less than the range inducing toxic effects in the *in vitro* model [ 24 ].

In the current study, no accumulation of zoledronate was observed in the dental tissues. A proportion of zoledronate is released during the bone remodeling process and the drug is likely to be exposed to the environment [ 25 ]. Basso *et al*. [ 26 ] showed that zoledronate had a significant effect on the reduction of the number of viable cells as well as the viability of epithelial cells (by 70%) and fibroblasts (by 40%). This revealed the toxicity of zoledronate for these cells. Agis *et al*. [ 14 ] evaluated the effects of zoledronate with a concentration of 30 μM in fibroblast cultures. Their results demonstrated the reduction of cell viability and proliferation in the fibroblasts. The differences in the results of various studies can be due to the different concentrations of zoledronate used in them. This is consistent with some studies that have reported the dose-dependent toxicity of bisphosphonate medications [ 15 ].

In this study, the evaluation of the cellular morphology of the dental and periodontal tissues did not show any significant differences between the treated and control groups. The results of the histological evaluation of the dental pulp and the surrounding tissues showed no significant differences between the control and treated groups. According to Basso *et al*. [ 26 ], the incidence of cell death was evident for some epithelial cells and fibroblasts with the separation of the glass substrate in the zoledronate-treated patients, although most of the cells adhered to the substrate and showed significant morphological changes. They also indicated that zoledronate (with a concentration of 5μM) was toxic for epithelial cells and fibroblasts [ 26 ]. These results cannot be generalized to clinical conditions. In addition, the effects of bisphosphonates in the oral cavity remain unknown. *In vitro* and *in vivo* studies have demonstrated that bispho-sphonates prevent the function of endothelial cells [ 27 ]. By reducing the blood flow, bisphosphonates play an antiangiogenic role in the necrosis areas [ 28 ]. Another cause of the osteonecrosis of the jaw is the function of bacteria. Bisphosphonates induce the apoptosis of the osteoclasts activated by tooth extraction or periodontal surgical processes [ 28 ]. Osteoclasts have adverse effects on the wound healing process by reducing cytokines in the bone marrow and bone surfaces, increasing the risk of osteomyelitis and necrosis [ 29 ]. Both jaw bones have a high rate of remodeling due to their mechanical ability in mastication. It is assumed that bisphosphonates accumulate in the bone and are simultaneously toxic for the oral cavity. They prevent soft tissue healing by causing secondary infections under bone tissues [ 29 ].

Overall, intravenous bisphosphonates have harmful effects on dental pulp cells and other tissues. The drugs used in chemotherapy lead to the reduction of pulp cells and fibrosis. Alternative managements play an important role in vital pulp therapy. In the present study, there were no symptoms in the tissues around the teeth of the animals receiving zoledronate. However, further clinical studies are needed to clarify this result.

## Conclusion

According to the histological findings, the periodontal ligament space, the bone adjacent to the roots, and the dental pulp conditions were similar in both groups. Osteonecrosis of the jaw did not develop in the rats that took bisphosphonates after intraligamental injection.

## Acknowledgement

This article is based on Mehdi Anaraki’s postgraduate thesis (No. 565). It was supported by the Vice Chancellor for Research of Mashhad University of Medical Sciences, Mashhad, Iran.

## Conflict of Interest

The authors declare that they have no conflict of interest.

## References

[1] Froughreyhani M, Salem Milani A, Barakatein B, Shiezadeh V ( 2013). Treatment of strip perforation using root mta: a case report. Iran Endod J.

[2] Rozental TD, Vazquez MACA, Chacko AT, Ayogu N, Bouxsein ML ( 2009). Comparison of radiographic fracture healing in distal radius for patients on and off bisphosphonate therapy. J Hand Surg Am.

[3] Kates SL, Ackert-Bicknell CL ( 2016). How do bisphosphonates affect fracture healing?. Injury.

[4] Meganck JA, Begun DL, McElderry JD, Swick A, Kozloff KM, Goldstein SM, et al ( 2013). Fracture healing with alendronate treatment in the Brtl/+ mouse model of osteogenesis imperfecta. Bone.

[5] Marx RE, Sawatari Y, Fortin M, Broumand V ( 2005). Bisphosphonate-induced exposed bone (osteonecrosis/ osteopetrosis) of the jaws: risk factors, recognition, prevention, and treatment. J Oral Maxillofac Surg.

[6] Smith GN, Walton RE ( 1983). Periodontal ligament injection: ditribution of injected solutions. Oral Surg Oral Med Oral Pathol.

[7] Nusstein JCE, Reader A, Beck M, Weaver J ( 2005). Anesthetic effectiveness of the supplemental intraligamentary injection, administered with a computer-controlled local anesthetic delivery system, in patients with irreversible pulpitis. J Endod.

[8] Maahs MP, Azambuja AA, Campos MM, Salum FG, Cherubini K ( 2011). Association between bisphosphonates and jaw osteonecrosis: a study in Wistar rats. Head Neck.

[9] Chaplet M, Detry C, Deroanne C, Fisher LW, Castronovo V, Bellahcene A ( 2004). Zoledronic acid up-regulates bone sialoprotein expression in osteoblastic cells through Rho GTPase inhibition. Biochem J.

[10] Harokopakis-Hajishengallis E ( 2007). Physiologic root resorption in primary teeth: molecular and histological events. J Oral Sci.

[11] Golomb G, Langer R, Schoen FJ, Smith MS, Choi YM, Levy RJ ( 1986). Controlled release of diphosphonate to inhibit bioprosthetic heart valve calcification: dose-response and mechanistic studies. J Control Release.

[12] Green JR, Müller K, Jaeggi KA ( 1994). Preclinical pharmacology of CGP 42′446, a new, potent, heterocyclic bisphosphonate compound. J Bone Miner Res.

[13] Gronich N, Rennert G ( 2013). Beyond aspirin-cancer prevention with statins, metformin and bisphosphonates. Nat Rev Clin Oncol.

[14] Harokopakis-Hajishengallis E ( 2007). Physiologic root resorption in primary teeth: molecular and histological events. J Oral Sci.

[15] Hill LF, Lumb GA, Mawer EB, Stanbury SW ( 1973). Indirect i- nhibition of the biosynthesis of 1, 25-dihydroxycholecal-ciferol in rats treated with a diphosphonate. Clin Sci.

[16] Hillner BE, Ingle JN, Chlebowski RT, J Gralow, Yee GC, Janjan NA, et al ( 2003). American society of clinical oncology 2003 update on the role of bisphosphonates and bone health issues in women with breast cancer. J Clin Oncol.

[17] Hillner BE, Weeks JC, Desch CE, Smith TJ ( 2000). Pamidronate in prevention of bone complications in metastatic breast cancer: a cost-effectiveness analysis. J Clin Oncol.

[18] Hiraga T, Ninomiya T, Hosoya A, Nakamura H ( 2010). Administration of the bisphosphonate zoledronic acid during tooth development inhibits tooth eruption and formation and induces dental abnormalities in rats. Calcif Tissue Int.

[19] Allam E, Allen Mr, Chu TM, Ghoneima A, Jack Windsor L ( 2011). In vivo effects of zoledronic acid on oral mucosal epithelial cells. Oral Dis.

[20] Hollander W, Prusty S, Nagraj S, Kirkpatrick B, Paddock J, Colombo M ( 1978). Comparative effects of cetaben (PHB) and dichlormethylene diphosphonate (Cl2MDP) on the development of atherosclerosis in the cynomolgus monkey. Atherosclerosis.

[21] Hortobagyi GN, Theriault RL, Porter L, Blayney D, Lipton A, Sinoff C, et al ( 1996). Efficacy of pamidronate in reducing skeletal complications in patients with breast cancer and lytic bone metastases. protocol 19 aredia breast cancer study group. N Engl J Med.

[22] Pourgonabadi S, Mousavi SH, Tayarani-Najaran Z, Ghorbani A ( 2018). Effect of zoledronate, a third-generation bisphosphonate, on proliferation and apoptosis of human dental pulp stem cells. Can J Physiol Pharmacol.

[23] Pourgonabadi S, Ghorbani A, Tayarani Najarn Z, Mousavi SH ( 2018). In vitro assessment of alendronate toxic and apoptotic effects on human dental pulp stem cells. Iran J Basic Med Sci.

[24] Jowsey J, Riggs BL, Kelly PJ, Hoffman DL, Bordier P ( 1971). The treatment of osteoporosis with disodium ethane-1-hydroxy-1, 1-diphosphonate. J Lab Clin Med.

[25] Atkin I, Ornoy A, Pita JC, Muniz OE, Agundez A, Castiglione G, et al ( 1988). EHDP‐induced rachitic syndrome in rats is not reversed by vitamin D metabolites. Anat Rec.

[26] Basso FG, Pansani TN, de Oliveira CF, Turrioni APS, Soares DG, Hebling J, et al ( 2013). Cytotoxic effects of zoledronic acid on human epithelial cells and gingival fibroblasts. Braz Dent J.

[27] Kilickap S, Ozdamar Y, Altundag MK, Dizdar O ( 2008). A case report: zoledronic acid-induced anterior uveitis. Med Oncol.

[28] Kobayashi YT, Hiraga A, Ueda L, Wang M, Matsumoto-Nakano K, Hata H, et al ( 2010). Zoledronic acid delays wound healing of the tooth extraction socket, inhibits oral epithelial cell migration, and promotes proliferation and adhesion to hydroxyapatite of oral bacteria, without causing osteonecrosis of the jaw, in mice. J Bone Miner Metab.

[29] Kramsch DM, Chan CT ( 1978). The effect of agents interfering with soft tissue calcification and cell proliferation on calcific fibrous-fatty plaques in rabbits. Circ Res.

